# The Presence of Ankylosing Spondylitis and the Incidence of Subsequent External Eye Diseases: A Population-Based Cohort Study

**DOI:** 10.3390/ijerph192316296

**Published:** 2022-12-05

**Authors:** Chia-Yi Lee, Hung-Chi Chen, Jing-Yang Huang, Chieh-Hung Yen, Yih-Shiou Hwang, Chao-Kai Chang, Shun-Fa Yang

**Affiliations:** 1 Institute of Medicine, Chung Shan Medical University, Taichung 40201, Taiwan; 2 Nobel Eye Institute, Taipei 100008, Taiwan; 3 Department of Ophthalmology, Jen-Ai Hospital Dali Branch, Taichung 41265, Taiwan; 4 Department of Ophthalmology, Chang Gung Memorial Hospital at Linkou, Taoyuan City 333423, Taiwan; 5 College of Medicine, Chang Gung University, Taoyuan City 333323, Taiwan; 6 Center for Tissue Engineering, Chang Gung Memorial Hospital, Linkou 33302, Taiwan; 7 Department of Medical Research, Chung Shan Medical University Hospital, Taichung 40201, Taiwan; 8 Graduate Institute of Biomedical Engineering, Chang Gung University, Taoyuan City 333323, Taiwan; 9 Department of Optometry, Da-Yeh University, Chunghua 51500, Taiwan

**Keywords:** ankylosing spondylitis, dry eye disease, keratopathy, population-based, inflammation

## Abstract

We aimed to survey the risk of external eye diseases in those with ankylosing spondylitis (AS) via the National Health Insurance Research Database (NHIRD) in Taiwan. We conducted a retrospective cohort study, and subjects diagnosed with AS were selected from the NHIRD. Then, the AS patients were matched 1:1 by propensity-score matching (PSM) to non-AS patients, and a total of 6754 participants were included in the AS and non-AS groups. The main outcomes were regarded as the occurrence of dry eye disease (DED), superficial keratopathy and corneal ulcers. We used Cox proportional hazard regression to yield the adjusted hazard ratios (AHR) with 95% confidence intervals (CI) between the AS and non-AS groups. There were 709 and 408 external eye disease events that occurred in the AS and non-AS groups after a tracking interval of up to 17 years. The incidence of all external eye diseases was significantly higher in the AS group than the non-AS group (AHR: 1.826, 95% CI: 1.616–2.063, *p* < 0.0001). Additionally, the rates of DED (AHR: 1.973, 95% CI: 1.701–2.290, *p* < 0.0001) and superficial keratopathy (AHR: 1.593, 95% CI: 1.347–1.883, *p* < 0.0001) were significantly higher in the AS group than the non-AS group. In the sub-group analyses, the possibility of any external eye disease (*p* = 0.0030) and DED (*p* = 0.0386) was decreased in the older age group compared to those in the middle-aged group. In conclusion, AS is significantly correlated to subsequent external eye diseases, mainly the DED and superficial keratopathy.

## 1. Introduction

Ankylosing spondylitis (AS) is an inflammatory and autoimmune disease that causes spinal and sacroiliac inflammation [1,2]. In previous research, the prevalence of AS was approximately 0.5 percent in the United States [3]. Clinical symptoms of AS include low back pain, reduced spinal mobility, and morning stiffness [4]. In a laboratory exam, the HLA-B27 is a prominent immune marker for AS, and approximately 85–90 percent of AS patients have a positive result [4,5]. For the treatment of AS, local glucocorticoid injection, non-steroid anti-inflammatory drugs and tumor necrosis factor-alpha inhibitors can be applied, although only 60 percent of patients showed an acceptable response after therapy [2,6].

The existence of AS Is associated with several ophthalmic diseases [7]. Due to the inflammatory nature of AS, acute anterior uveitis is the most prevalent extra-articular manifestation of AS, which accounts for approximately 30 percent of AS patients [8]. In another study, the history of any uveitis was observed in 32 percent of the AS population [9]. Additionally, choroidoretinitis and panuveitis were observed in the presence of AS, although they were less common than iridocyclitis [10]. The other ophthalmic diseases related to the AS are papillitis, retinal vasculitis, vitritis, cystoid macular edema, pars plana exudate and epiretinal membranes [11,12].

Some research has reported the possibility of a relationship between AS and external eye disease [10,13]. A previous study showed that patients with AS had a weaker cornea with lower corneal hysteresis and a thinner central corneal thickness [13]. Additionally, conjunctivitis and scleritis have been demonstrated in individuals diagnosed with AS [10,14]. Nevertheless, there are few relative studies that investigate the time sequence of AS and follow external eye diseases with adequate patient numbers. Since both the AS and external eye diseases, such as DED and corneal disorders, are characterized by inflammatory responses [3,15,16], a potential relationship may exist between them that needs further validation.

Consequently, we aimed to evaluate the correlation between the AS and subsequent external eye disease via the utilization of the National Health Insurance Research Database (NHIRD) in Taiwan. The external eye diseases included in the current study were DED, superficial keratopathy and corneal ulcers. 

## 2. Materials and Methods

### 2.1. Database Details 

The NHIRD store claimed data from the National Health Insurance of approximately all of Taiwan’s citizens, and the duration of data accessibility ranged from 1 January 2000 to 31 December 2018. For the current study, we accessed data from the Longitudinal Health Insurance Database 2000 (LHID 2000), which is a sub-database of NHIRD that preserves the medical records of approximately 2 million subjects who were randomly sampled from the NHIRD registry in the year 2000, and those data were connected from 1 January 2000 to 31 December 2018. The ninth and tenth revisions of the International Classification of Diseases were used for disease diagnosis in the NHIRD/LHID 2000, depending on the year of diagnosis. The NHIRD/LHID 2000 database system also provided demography, time of outpatient department (OPD) visit, codes of department, codes of exam and ATC codes of medications. 

### 2.2. Participant Selection 

**Figure 1:**
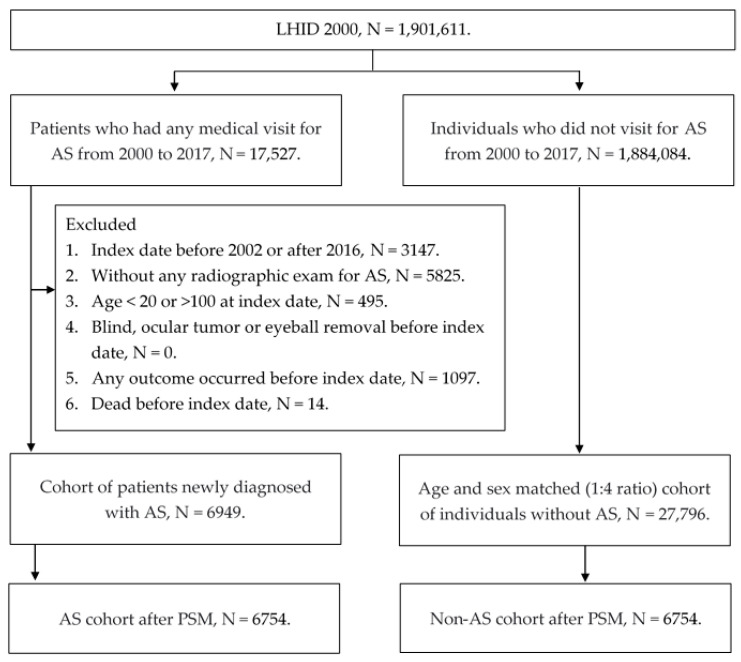
The flowchart of participant selection. N: number, AS: ankylosing spondylitis, PSM: propensity score-matching.

Participants were defined as having AS if they fulfilled the following inclusion criteria: (1) the receipt of AS diagnostic codes, (2) the arrangement of a spine X-ray, computed tomography or magnetic resonance imaging exam, (3) the arrangement of a HLA-B27 laboratory exam and (4) the diagnosis of AS was created by a rheumatologist. The index date was defined as 90 days after the first AS diagnosis. On the other hand, the following exclusion criteria were used to make the general condition of the study population more homogenous: (1) the index date occurred before 2002 or after 2016, (2) age was less than 20 or more than 100 at the index date, (3) the receipt of diagnoses related to blindness, an ocular tumor or eyeball removal that occurred before the index date, (4) any outcome that occurred before the index date and (5) died before the index date. The reason to exclude the patients with index dates before 2002 or after 2016 is to ensure a sufficient time period for both the follow-up and the exclusion of previous outcome achievement. After the above selection, each patient diagnosed with AS was matched to four non-AS individuals via age and gender, and then the propensity-score match (PSM) according to demography and specific systemic diseases was performed between the two populations in a 1:1 ratio. Of note, if a patient with AS could not be matched to one non-AS individual via PSM, this subject would be discarded from the current study. A total of 6754 participants were finally enrolled in the AS group and the non-AS group. The flow chart of participant selection is shown in Figure 1. 

### 2.3. Main Outcome Definition

There are three main outcomes in this study: DED, superficial keratopathy and corneal ulcers. The definition of outcome achievement included the following conditions: (1) receipt of diagnoses related to DED, superficial keratopathy and corneal ulcers, (2) the arrangement of a corneal fluorescein stain examination, (3) the prescription of associated medications for DED, superficial keratopathy and corneal ulcers and (4) the above managements were performed by an ophthalmologist. For more details, the diagnoses that were regarded as superficial keratopathy include unspecified superficial keratitis, macular keratitis, filamentary keratitis, photokeratitis, punctate keratitis, unspecified keratoconjunctivitis, exposure keratoconjunctivitis, neurotrophic keratoconjunctivitis, phlyctenular keratoconjunctivitis, vernal keratoconjunctivitis and recurrent erosion of the cornea. Additionally, the medications for DED were defined as artificial tears, and the medications for superficial keratopathy and corneal ulcer were regarded as antibiotic eyedrops. In addition, the “unspecified disorder of cornea” was not included in our main outcome to avoid overestimation of cases.

### 2.4. Demographic Variables and Comorbidities

Apart from the main outcomes, we also considered the influence of year of enrollment, age, gender, urbanization, educational level and the following systemic diseases in the multi-variable analyses to erase the possible confounders: hypertension, diabetes mellitus (DM), end-stage renal disease (ESRD), ischemic heart diseases, cerebrovascular disease, rheumatoid arthritis, systemic lupus erythematous and other inflammatory diseases. The “other inflammatory diseases” mainly included enteropathic arthropathies, gout, crystal arthropathies, osteoarthritis, polyarteritis nodosa, dermatopolymyositis, systemic sclerosis, inflammatory bowel disease and psoriatic arthritis. We longitudinally trailed the data of each patient from the index date to the date of the main outcome complement, individual withdrawal from the National Health Insurance program or to the end of NHIRD/LHID 2000, which means 31 December 2018.

### 2.5. Statistical Analysis

The SAS version 9.4 (SAS Institute Inc., Cary, NC, USA) was adopted for the statistical analyses in this study. Firstly, we used the descriptive analysis to reveal the distribution of basic manifestations between the AS and non-AS groups. The absolute standardized difference (ASD) between the AS and non-AS groups was calculated, and an ASD value less than 0.1 was regarded as statistically insignificant. After that, the incidence rate was produced via Poisson regression, and the adjusted hazard ratios (AHR) with corresponding 95% confidence intervals (CI) of the main outcomes between the AS and non-AS groups were conducted through the Cox proportional hazard regression, which incorporated both the effect of demographic data and systemic comorbidities. Concerning the sub-group analyses, we stratified the AS and non-AS populations based on age and gender, and then the Cox proportional hazard regression was applied again for the AHR and 95% CI. Additionally, the trend among different age and gender sub-groups was analyzed via the interaction test. We painted the Kaplan–Meier curve to demonstrate the cumulative incidence of each external eye disease between the AS and non-AS groups and utilized the log-rank test to evaluate the significance between the two groups. The statistical significance was defined as *p* < 0.05, and a *p*-value less than 0.0001 was revealed as *p* < 0.0001.

## 3. Results

The baseline characteristics of the AS and non-AS groups are shown in Table 1. Age and gender were nearly identical between the two groups due to the PSM process (both ASD = 0.0000). The distribution of the remaining demography and the systemic co-morbidities also demonstrated insignificant differences between the two groups after PSM (all the ASD < 0.100) (Table 1).

**Figure 2:**
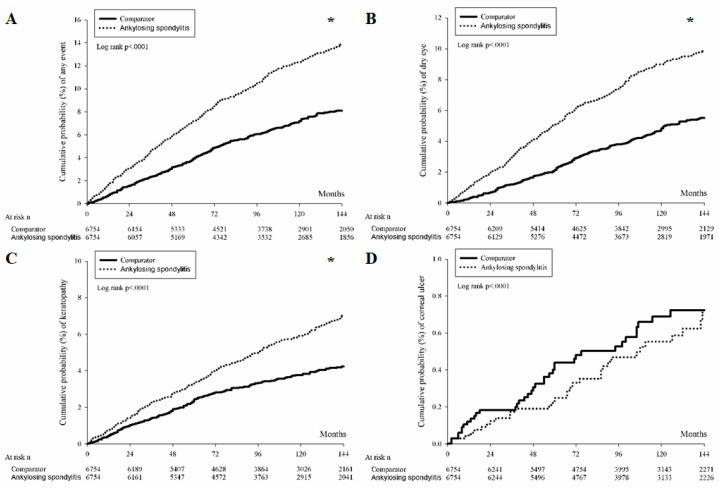
The cumulative incidence of external eye diseases between the ankylosing spondylitis and control groups. (**A**) The cumulative incidence of all external eye diseases. (**B**) The cumulative incidence of dry eye disease. (**C**) The cumulative incidence of superficial keratopathy. (**D**) The cumulative incidence of corneal ulcers. * denotes significant difference between groups.

A total of 709 and 408 external eye diseases occurred in the AS and non-AS groups after a tracking interval of up to 17 years. After considering the effect of each potential confounder, the incidence of all external eye diseases was significantly higher in the AS group than in the non-AS group (AHR: 1.826, 95% CI: 1.616–2.063, *p* < 0.0001) (Table 2). If we analyzed each external eye disease separately between the two groups, the incidence of DED (AHR: 1.973, 95% CI: 1.701–2.290, *p* < 0.0001) and superficial keratopathy (AHR: 1.593, 95% CI: 1.347–1.883, *p* < 0.0001) were significantly higher in the AS population compared to the non-AS counterpart. However, the incidence of corneal ulcers was similar between the two groups (*p* = 0.4598) (Table 2). The Kaplan–Meier curve demonstrated significantly higher cumulative probabilities of DED and superficial keratopathy in the AS group (both *p* < 0.0001) (Figure 2).

In the sub-group analyses, the incidence of DED and keratopathy were significantly higher in the patients with AS and younger than 70 years old, while both gender sub-groups showed a higher rate of DED and keratopathy with AS (all the lower limit of 95% CI was larger than 1) (Table 3). The interaction test, on the other hand, found that the incidences of any external eye disease (*p* = 0.0030) and DED (*p* = 0.0386) were lower in the older population than in the population of middle age (Table 3).

## 4. Discussion

In the current study, the overall incidence of external eye diseases was significantly higher in the AS group compared to the non-AS group. Additionally, the higher incidence of external eye diseases mainly resulted from the elevated DED and superficial keratopathy episodes. On the other hand, the correlation between AS and any of the external eye diseases decreased in the older population compared to the middle-aged population.

The AS has been proposed as both an inflammatory and an autoimmune disease [1]. The inflammatory pathway, including interleukin-17/23 and tumor necrosis factor receptors, was involved in the pathogenesis of AS, according to previous studies [3]. Additionally, mucosal inflammation in the gut developed in up to 70% of individuals with AS, which showed some correlation to the microbiome [17]. Regarding the autoimmune disease aspect, an article demonstrated the enhancement of the HLA-B27-related pathway and type 3 immunity in patients with AS [18,19]. In addition, other HLA families, such as HLA-B40, HLA-B51, HLA-B7, HLA-A2 and HLA-DPB1, were included in the pathogenesis of AS [6]. On the other hand, anti-CD74 autoantibodies were found in patients diagnosed with AS [20]. Other autoimmune diseases, such as arthritis and enthesitis, also correlate with the development of AS [6]. For external eye diseases, the DED has been established as an inflammatory disease in previous publications [15]. The release of inflammatory markers, such as matrix metalloproteinase-9, was observed in DED [21], which would contribute to the damage of goblet cells, increased hyperosmolarity of the ocular surface, and the subsequent instability of the tear film, thus, forming a vicious cycle [15]. For the treatment of DED, certain anti-inflammatory agents, such as steroids and cyclodporine, can be applied to treat DED, which reveal favorable outcomes on both signs and symptoms [22,23]. Regarding corneal disorders, superficial keratitis can result from infection, trauma or an inflammatory response [24]. Additionally, some of the DED cases would manifest with superficial keratopathy [24]. The etiology of corneal ulcers is mainly of infectious origin [16,25], but the micro-organism will also cause the ocular surface inflammation, and steroids can be applied to retard the inflammation under the cover of antibiotic agents [26]. Since the existence of AS is correlated with certain types of uveitis, which belong to ocular inflammation disorders [8,27], we speculated that external eye diseases with inflammatory reactions may be triggered by AS. This concept was supported by the findings of the current study, at least partially.

Our analyses demonstrated that the AS is associated with the development of the following external eye diseases, mainly the DED and superficial keratopathy. To our knowledge, there has not been a lot of research in this field. Additionally, we controlled the distribution of confounders between the two groups via PSM, and we considered the effect of several confounders in the Cox proportional hazard regression. Consequently, the AS is more likely an independent risk factor for the DED and superficial keratopathy. The DED had been proven to be associated with several systemic inflammatory diseases in which the rheumatic arthritis, systemic lupus erythematous and Sjogren syndrome would cause a higher incidence of DED development than the normal population [7]. Consequently, it is reasonable to expect a higher rate of DED in the AS population. Noteworthy, the steroid is used for AS control in certain conditions [28], and the steroid should theoretically decrease the incidence or severity of DED. Although we did not include the steroid in the multi-variable analysis, the AHR of DED was still higher in the AS group, which implies the prominent effect of AS. In addition to DED, the superficial keratopathy, especially the keratoconjunctivitis, is also associated with an inflammatory reaction [29]. Some systemic inflammatory diseases, such as psoriatic arthritis, could contribute to a higher incidence of specific superficial keratopathy [16]. Thus, the higher incidence of superficial keratopathy in the AS group can be explained by the similar etiology of DED. On the other hand, the incidence of corneal ulcers was similar between the AS and non-AS populations in the multi-variable analyses. Actually, the numbers of corneal ulcer events were numerically lower in the AS group than in the non-AS group. The possible reason is that the number of corneal ulcers is far less than the number of DED and superficial keratopathy events, which may lead to statistical bias. Additionally, the corneal ulcer is more of an infectious disease than an inflammatory disease [16], so the inflammation effect of AS may not be significant enough to cause corneal ulcer development.

In the sub-group analyses, older AS individuals exhibited a lower probability of external eye disease development compared to the middle-aged population. If we separated each external eye disease, the distribution of DED in different age sub-groups would lead to such a condition. Despite the fact that age is a prominent risk factor for DED [22], the relatively few patient numbers in the 70–100 year-old sub-group may let the statistical power diminish. On the other hand, the AS is more prevalent in the middle-aged population, approximately 30 years old, and the inflammatory back pain frequently develops in patients under 40 [17,28]. Accordingly, we speculate that the AS that occurred in the middle-aged population has the strongest inflammatory response and contributes to a higher incidence of subsequent DED compared to the non-AS group. For the risk of superficial keratopathy, a trend toward decline that is similar to the incidence of DED was found, although the change was marginally significant. The trend of corneal ulcer development did not change with different age groups, which is in accordance with the insignificant difference between the whole AS and non-AS population. In addition to the trend, the AHR of both DED and superficial keratopathy in AS patients was significantly higher than that of non-AS patients from age 20 to 70, which further illustrates the universal effect of AS on these two external eye diseases. Although AS was more common in men [28], the gender sub-group analyses did not find a significant difference between the two genders, and both the DED and superficial keratopathy revealed higher rates in AS patients of both genders. This finding corresponds to the analyses of the whole population.

For the epidemiological aspect, AS is a prevalent inflammatory disease worldwide, and the prevalence of AS is approximately 1.4 percent in the Caucasian population and approximately 0.06 percent in the non-Caucasian population [28]. In the northern Arctic communities, the prevalence of AS can reach 1.61 percent, according to previous studies [30]. In these AS patients, the mortality elevated to approximately 1.6 folds compared to the general population [30]. The DED is another disease that affects the majority of humans. In previous research, the prevalence of DED was approximately 6.8 percent in the general population and rose to 22.8 percent in female patients older than 75 years old [22]. According to another article, the prevalence of DED was highest in South-East Asia [23]. Except for the ocular irritation caused by DED, the DED can lead to visual impairment and decreased quality of life [22]. Moreover, the DED is associated with depressive disorder, thus, it can also affect the psychological side of human health [31]. Since both AS and DED influence a large number of patients, and the impact of DED on humans is both physical and mental, any relationship between AS and DED or other external eye diseases should be illustrated.

There are some limitations presented in the current study. Firstly, due to the claimed database nature of our study, we cannot access certain important issues, which include the radiographic finding of AS, the clinical symptoms of AS, the treatment outcome of AS, the types of DED, the severity of external eye diseases, the results of fluorescein stain and slit lamp examination and the therapeutic response of external eye diseases. Due to the design of NHIRD/LHID 2000, the activity of AS and severity of AS cannot be evaluated, and the duration of AS is also unavailable because we only enrolled newly diagnosed AS cases. Additionally, we cannot divide the outcome into more delicate categories with additional discussion for them because of the design of NHIRD/LHID 2000. Although we adjusted the effects of rheumatoid arthritis and systemic lupus erythematous in the Cox proportional hazard regression and proved that AS is an independent risk factor for DED and superficial keratopathy even under the influence of the above two diseases, the activity of the two diseases cannot be evaluated in NHIRD/LHID 2000, which may alter the results of our study. Additionally, the tracking system of NHIRD/LHID 2000 only allows us to find one main outcome, at which point the tracking would be stopped, thus, we cannot know whether a participant develops superficial keratopathy after the DED diagnosis. On the other hand, the retrospective design of our study would retard the homogeneity of our study population compared to a prospective study, despite the fact that we conducted PSM to decrease the difference between the two groups. Additionally, we lost more than 50 percent of the cases during the exclusion process, which may lead to some bias. However, those we excluded were due to an inadequate follow-up period or the absence of an image survey, thus, we think this exclusion is acceptable for the accuracy of diagnosis and follow-up. Additionally, the patient number was 6754 in both groups, which was not inferior to the previous population-based study [32], thus, the statistical power might be adequate.

## 5. Conclusions

In conclusion, the existence of AS is correlated with a higher rate of external eye diseases in the population aged 20–70 years old. Furthermore, the AS affects mainly the development of DED and superficial keratopathy but not the more severe corneal ulcer. Consequently, additional ophthalmic care may be needed for the AS patients if any ocular symptoms occur. Furthermore, a large-scale prospective study to evaluate whether AS would alter the therapeutic response of DED and superficial keratopathy is mandatory.

## Figures and Tables

**Table 1:** Baseline characteristics between the two groups.

Characteristics	Non-AS Group N = 6754	AS Group N = 6754	ASD
Year of index			0.0000
2002–2006	2992 (44.30%)	2966 (43.91%)	
2007–2011	1959 (29.01%)	1989 (29.45%)	
2012–2017	1803 (26.70%)	1799 (26.64%)	
Sex			0.0000
Male	4427 (65.55%)	4427 (65.55%)	
Female	2327 (34.45%)	2327 (34.45%)	
Age at index			0.0000
20–30	1786 (26.44%)	1748 (25.88%)	
30–39	1551 (22.96%)	1545 (22.88%)	
40–49	1377 (20.39%)	1359 (20.12%)	
50–59	1074 (15.90%)	1082 (16.02%)	
60–69	547 (8.10%)	561 (8.31%)	
70–100	419 (6.20%)	459 (6.80%)	
Urbanization			0.0219
Urban	4000 (59.22%)	3948 (58.45%)	
Sub-urban	2186 (32.37%)	2216 (32.81%)	
Rural	568 (8.41%)	590 (8.74%)	
Education			0.0000
Elementary school or below	1763 (26.10%)	1759 (26.04%)	
Junior high school	1152 (17.06%)	1181 (17.49%)	
Senior high school	2982 (44.15%)	2953 (43.72%)	
University or above	857 (12.69%)	861 (12.75%)	
Co-morbidities			
Hypertension	978 (14.48%)	1019 (15.09%)	0.0171
DM	338 (5.00%)	398 (5.89%)	0.0391
ESRD	39 (0.58%)	41 (0.61%)	0.0039
Ischemic heart diseases	161 (2.38%)	211 (3.12%)	0.0452
Cerebrovascular disease	65 (0.96%)	87 (1.29%)	0.0309
Rheumatoid arthritis	14 (0.21%)	10 (0.15%)	0.0141
Systemic lupus erythematous	49 (0.18%)	10 (0.14%)	0.0024
Other inflammatory diseases	60 (0.89%)	60 (0.89%)	0.0000

AS: ankylosing spondylitis, N: number, ASD: absolute standardized difference, DM: diabetes mellitus, ESRD: end-stage renal disease.

**Table 2:** Incidence risk of main outcome between propensity score matched ankylosing spondylitis and non-ankylosing spondylitis groups.

Event	Non-AS N = 6754	AS N = 6754	*p* Value
**Any outcome**			
Follow up person months	696,943	666,896	
New case	408	709	
Incidence rate † (95% CI)	0.59 (0.53–0.65)	1.06 (0.99–1.14)	
Crude Relative risk (95% CI)	Reference	1.802 (1.595–2.035)	
AHR (95% CI)	Reference	1.826 (1.616–2.063)	<0.0001 *
**Dry eye**			
Follow up person months	710,667	685,762	
New case	267	504	
Incidence rate † (95% CI)	0.38 (0.33–0.42)	0.73 (0.67–0.80)	
Crude Relative risk (95% CI)	Reference	1.947 (1.678–2.258)	
AHR (95% CI)	Reference	1.973 (1.701–2.290)	<0.0001 *
**Keratopathy**			
Follow up person months	712,411	697,502	
New case	225	351	
Incidence rate † (95% CI)	0.32 (0.28–0.36)	0.50 (0.45–0.56)	
Crude Relative risk (95% CI)	Reference	1.586 (1.341–1.875)	
AHR (95% CI)	Reference	1.593 (1.347–1.883)	<0.0001 *
**Corneal ulcer**			
Follow up person months	729,389	726,261	
New case	42	34	
Incidence rate † (95% CI)	0.06 (0.04–0.08)	0.05 (0.03–0.07)	
Crude Relative risk (95% CI)	Reference	0.813 (0.517–1.278)	
AHR (95% CI)	Reference	0.805 (0.512–1.266)	0.4598

AS: ankylosing spondylitis, N: number, DED: dry eye disease, AHR: adjusted hazard ratio, CI: confidence interval, * denotes significant difference between the two groups after adjusting age, sex, urbanization, education level and co-morbidities, † incidence rate: per 1000 person-month.

**Table 3:** Stratified analysis via age and gender in ankylosing spondylitis population.

Parameters (AHR ± 95% CI)	Any Event	DED	Keratopathy	Corneal Ulcer
**Age**				
20–30	1.340 (1.045–1.719)	1.618 (1.122–2.333)	1.262 (0.929–1.715)	0.571 (0.248–1.314)
30–39	1.857 (1.492–2.312)	2.064 (1.561–2.728)	1.555 (1.157–2.090)	0.784 (0.327–1.881)
40–49	1.900 (1.566–2.305)	2.051 (1.636–2.571)	1.647 (1.247–2.175)	0.681 (0.264–1.757)
50–59	2.053 (1.725–2.443)	2.204 (1.811–2.681)	1.844 (1.428–2.382)	0.809 (0.323–2.024)
60–69	1.632 (1.308–2.038)	1.458 (1.117–1.904)	1.464 (1.064–2.015)	1.746 (0.756–4.030)
70–100	1.068 (0.779–1.464)	1.387 (0.961–2.001)	0.836 (0.536–1.306)	1.204 (0.348–4.163)
*p* for interaction	0.0030	0.0386	0.0997	0.4130
**Sex**				
Male	1.737 (1.530–1.972)	1.956 (1.667–2.294)	1.466 (1.232–1.744)	0.975 (0.617–1.540)
Female	1.711 (1.515–1.933)	1.812 (1.572–2.089)	1.519 (1.277–1.809)	0.702 (0.379–1.301)
*p* for interaction	0.9468	0.6222	0.8149	0.3192

DED: dry eye disease, AHR: adjusted hazard ratio, CI: confidence interval.

## Data Availability

Due to the policy of the National Health Insurance Administration in Taiwan, the raw data of this study is not available.
